# 2,4-Diamino-6-methyl-1,3,5-triazine ethanol solvate

**DOI:** 10.1107/S1600536808000159

**Published:** 2008-01-09

**Authors:** Zun-Hong Xiao

**Affiliations:** a School of Physics and Chemistry Science, Guizhou Normal University, Guiyang, 550001, People’s Republic of China.

## Abstract

The crystal structure of the title compound, C_4_H_7_N_5_·C_2_H_6_O, is determined by extensive hydrogen bonding. A sequence of dimeric associations, formed by N—H(amino)⋯N(ring), connects the triazine rings into a mol­ecular tape. Mol­ecules are linked into a supra­molecular structure by N—H⋯O and O—H⋯O hydrogen bonds. The asymmetric unit consists of two formula units.

## Related literature

For general background, see: Sebenik *et al.* (1989 ▶); Tashiro & Oiwa (1981 ▶).
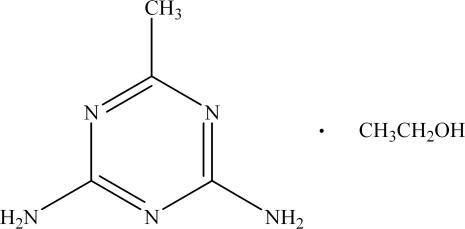


## Experimental

### 

#### Crystal data

C_4_H_7_N_5_·C_2_H_6_O
*M* *_r_* = 171.21
Triclinic, 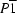

*a* = 8.3860 (6) Å
*b* = 9.1514 (6) Å
*c* = 11.9104 (9) Å
α = 88.703 (1)°
β = 87.614 (2)°
γ = 76.668 (2)°
*V* = 888.56 (11) Å^3^
*Z* = 4
Mo *K*α radiation
μ = 0.09 mm^−1^
*T* = 273 (2) K
0.34 × 0.26 × 0.21 mm

#### Data collection

Bruker SMART CCD area-detector diffractometer
Absorption correction: multi-scan (*SADABS*; Bruker, 2005 ▶) *T*_min_ = 0.975, *T*_max_ = 0.985
7627 measured reflections
3111 independent reflections
2619 reflections with *I* > 2σ(*I*)
*R*_int_ = 0.017

#### Refinement

*R*[*F* ^2^ > 2σ(*F* ^2^)] = 0.042
*wR*(*F* ^2^) = 0.118
*S* = 1.07
3111 reflections
223 parameters
H-atom parameters constrained
Δρ_max_ = 0.25 e Å^−3^
Δρ_min_ = −0.19 e Å^−3^

### 

Data collection: *SMART* (Bruker, 2002 ▶); cell refinement: *SAINT* (Bruker, 2002 ▶); data reduction: *SAINT*; program(s) used to solve structure: *SHELXS97* (Sheldrick, 2008 ▶); program(s) used to refine structure: *SHELXL97* (Sheldrick, 2008 ▶); molecular graphics: *ORTEP-3 for Windows* (Farrugia, 1997 ▶); software used to prepare material for publication: *WinGX* (Farrugia, 1999 ▶).

## Supplementary Material

Crystal structure: contains datablocks global, I. DOI: 10.1107/S1600536808000159/bq2061sup1.cif

Structure factors: contains datablocks I. DOI: 10.1107/S1600536808000159/bq2061Isup2.hkl

Additional supplementary materials:  crystallographic information; 3D view; checkCIF report

## Figures and Tables

**Table 1:** Hydrogen-bond geometry (Å, °)

*D*—H⋯*A*	*D*—H	H⋯*A*	*D*⋯*A*	*D*—H⋯*A*
N4—H4*B*⋯N7^i^	0.86	2.11	2.9666 (18)	171
N5—H5*D*⋯N8^ii^	0.86	2.19	3.0132 (19)	159
N5—H5*E*⋯O2^iii^	0.86	2.29	3.0071 (19)	142
N10—H10*A*⋯O2^iv^	0.86	2.10	2.9337 (18)	163
O2—H2⋯O1^v^	0.82	1.90	2.7185 (18)	174

Symmetry codes: (i) 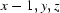; (ii) 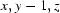; (iii) 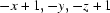; (iv) 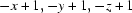; (v) 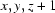.

**Fig. 1.**
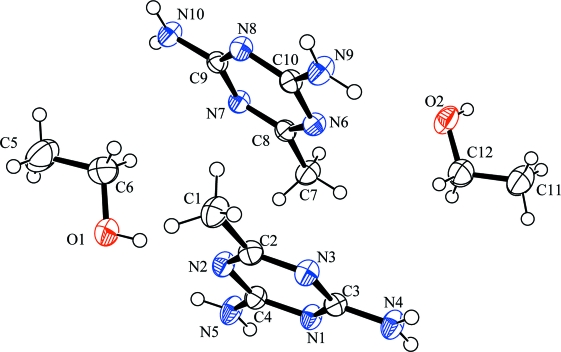
The molecular structure of (I) showing the atom-labeling scheme. Displacement ellipsoids are drawn at the 50% probability level.

## supplementary crystallographic information

### Comment

Triazine compounds are used in pharmaceutical industry as coupling agents for the synthesis of peptides and as side chain of antibiotics, as well as in formulating bactericides and fungicides. 2,4-Diamino-6-methyl-1,3,5-triazine (acetoguanamine) is used as an intermediate for pharmaceuticals and as a modifier and flexibilizer of formaldehyde resins (Sebenik *et al.*, 1989, Tashiro *et al.*, 1981).

The crystal structure of the title compound (Fig. 1) consists of triazine and solvate ethanol molecule. The amino groups are coplanar with the ring plane, the dihedral angle between the triazine ring (C2,N2,C4,N1,C3,N3) and the ring (C8,N7,C9,N8,C10,N6) is 12.73 (7)°. A lot of hydrogen bonds are observed (Table 1), each NH_2_ group acts as a donor in hydrogen bond with the ring nitrogen atoms of neighboring molecules, these contacts and the cross-linking interactions stabilize the crystal packing.

### Experimental

2,4-diamino-6-methyl-1,3,5-triazine (0.625 g, 0.05 mol) was added to a stirred solvent of ethanol (100 ml) at 50°C for 3 h. After cooling to room temperature, the mixture was filtered. The filtrate was set aside for one week to obtain colorless crystals.

### Refinement

Water H atoms were located in a difference Fourier map and refined as riding in their as-found positions relative to O atoms with *U*_iso_(H) = 1.2*U*_eq_(O). All other H atoms were placed in calculated positions and refined as riding, with C—H = 0.93–0.97 Å, N—H = 0.86 Å, and *U*_iso_(H) = 1.2–1.5 *U*_eq_(C,*N*).

### Crystal data

**Table:**

C_4_H_7_N_5_·C_2_H_6_O	*Z* = 4
*M**_r_* = 171.21	*F*_000_ = 368.0
Triclinic, *P*1	*D*_x_ = 1.280 Mg m^−^^3^
Hall symbol: -P 1	Mo *K*α radiation λ = 0.71073 Å
*a* = 8.3860 (6) Å	Cell parameters from 3111 reflections
*b* = 9.1514 (6) Å	θ = 1.7–25.0º
*c* = 11.9104 (9) Å	µ = 0.09 mm^−^^1^
α = 88.703 (1)º	*T* = 273 (2) K
β = 87.614 (2)º	Block, colorless
γ = 76.668 (2)º	0.34 × 0.26 × 0.21 mm
*V* = 888.56 (11) Å^3^	

### Data collection

**Table:**

Bruker CCD area-detector diffractometer	3111 independent reflections
Radiation source: fine-focus sealed tube	2619 reflections with *I* > 2σ(*I*)
Monochromator: graphite	*R*_int_ = 0.017
*T* = 273(2) K	θ_max_ = 25.0º
φ and ω scans	θ_min_ = 1.7º
Absorption correction: multi-scan(SADABS; Bruker, 2005)	*h* = −9→9
*T*_min_ = 0.975, *T*_max_ = 0.985	*k* = −10→10
7627 measured reflections	*l* = −12→14

### Refinement

**Table:**

Refinement on *F*^2^	Secondary atom site location: difference Fourier map
Least-squares matrix: full	Hydrogen site location: inferred from neighbouring sites
*R*[*F*^2^ > 2σ(*F*^2^)] = 0.042	H-atom parameters constrained
*wR*(*F*^2^) = 0.118	*w* = 1/[σ^2^(*F*_o_^2^) + (0.0597*P*)^2^ + 0.2811*P*] where *P* = (*F*_o_^2^ + 2*F*_c_^2^)/3
*S* = 1.07	(Δ/σ)_max_ < 0.001
3111 reflections	Δρ_max_ = 0.25 e Å^−^^3^
223 parameters	Δρ_min_ = −0.19 e Å^−^^3^
Primary atom site location: structure-invariant direct methods	Extinction correction: none

### Special details

**Table:**

Geometry. All e.s.d.'s (except the e.s.d. in the dihedral angle between two l.s. planes) are estimated using the full covariance matrix. The cell e.s.d.'s are taken into account individually in the estimation of e.s.d.'s in distances, angles and torsion angles; correlations between e.s.d.'s in cell parameters are only used when they are defined by crystal symmetry. An approximate (isotropic) treatment of cell e.s.d.'s is used for estimating e.s.d.'s involving l.s. planes.
Refinement. Refinement of *F*^2^ against ALL reflections. The weighted *R*-factor *wR* and goodness of fit *S* are based on *F*^2^, conventional *R*-factors *R* are based on *F*, with *F* set to zero for negative *F*^2^. The threshold expression of *F*^2^ > σ(*F*^2^) is used only for calculating *R*-factors(gt) *etc*. and is not relevant to the choice of reflections for refinement. *R*-factors based on *F*^2^ are statistically about twice as large as those based on *F*, and *R*- factors based on ALL data will be even larger.

### Fractional atomic coordinates and isotropic or equivalent isotropic displacement parameters (Å^2^)

**Table:**

	*x*	*y*	*z*	*U*_iso_*/*U*_eq_	
C1	0.0454 (2)	0.41963 (19)	0.15635 (15)	0.0388 (4)	
H1A	−0.0653	0.4778	0.1527	0.058*	
H1B	0.0892	0.3946	0.0817	0.058*	
H1C	0.1110	0.4770	0.1920	0.058*	
C2	0.04756 (19)	0.27874 (17)	0.22268 (13)	0.0290 (4)	
C3	−0.07229 (18)	0.14537 (17)	0.34835 (13)	0.0258 (3)	
C4	0.18337 (18)	0.04532 (17)	0.27722 (13)	0.0268 (3)	
C5	0.6644 (3)	0.2978 (3)	0.0124 (2)	0.0651 (6)	
H5A	0.7394	0.2012	0.0089	0.098*	
H5B	0.7212	0.3716	0.0348	0.098*	
H5C	0.6200	0.3244	−0.0602	0.098*	
C6	0.5286 (2)	0.2917 (2)	0.09584 (17)	0.0486 (5)	
H6A	0.5730	0.2628	0.1691	0.058*	
H6B	0.4548	0.3900	0.1019	0.058*	
C7	0.3737 (2)	0.18852 (18)	0.49621 (15)	0.0342 (4)	
H7A	0.4172	0.1132	0.4412	0.051*	
H7B	0.2683	0.1765	0.5241	0.051*	
H7C	0.4469	0.1780	0.5572	0.051*	
C8	0.35597 (18)	0.34072 (16)	0.44340 (12)	0.0254 (3)	
C9	0.47170 (18)	0.50943 (16)	0.34763 (12)	0.0244 (3)	
C10	0.20147 (18)	0.56977 (16)	0.39979 (12)	0.0255 (3)	
N1	0.05944 (15)	0.02890 (14)	0.34824 (11)	0.0279 (3)	
N2	0.18531 (16)	0.17023 (15)	0.21443 (11)	0.0299 (3)	
N3	−0.08554 (15)	0.27267 (14)	0.28518 (11)	0.0284 (3)	
N4	−0.20112 (16)	0.13534 (15)	0.41484 (11)	0.0323 (3)	
H4A	−0.1984	0.0563	0.4559	0.039*	
H4B	−0.2871	0.2079	0.4167	0.039*	
N6	0.20797 (15)	0.43256 (14)	0.44771 (11)	0.0273 (3)	
N7	0.49186 (15)	0.37128 (13)	0.39648 (10)	0.0268 (3)	
N8	0.32886 (15)	0.61343 (14)	0.34805 (11)	0.0274 (3)	
N9	0.05530 (15)	0.66659 (15)	0.40412 (12)	0.0336 (3)	
H9A	0.0442	0.7546	0.3742	0.040*	
H9B	−0.0276	0.6409	0.4368	0.040*	
N10	0.60412 (15)	0.54140 (15)	0.29698 (11)	0.0309 (3)	
H10A	0.5979	0.6276	0.2650	0.037*	
H10B	0.6959	0.4759	0.2962	0.037*	
O1	0.44204 (16)	0.18580 (16)	0.06094 (10)	0.0475 (4)	
H1	0.3607	0.1897	0.1024	0.071*	
C11	0.0989 (3)	0.1326 (3)	0.89654 (19)	0.0599 (6)	
H11A	0.1324	0.1124	0.9725	0.090*	
H11B	0.0321	0.0655	0.8769	0.090*	
H11C	0.0370	0.2344	0.8900	0.090*	
C12	0.2460 (2)	0.1100 (2)	0.81971 (17)	0.0495 (5)	
H12A	0.3036	0.0053	0.8237	0.059*	
H12B	0.2103	0.1310	0.7434	0.059*	
N5	0.31467 (16)	−0.06868 (15)	0.26948 (12)	0.0353 (3)	
H5D	0.3180	−0.1490	0.3091	0.042*	
H5E	0.3961	−0.0619	0.2249	0.042*	
O2	0.35702 (16)	0.19983 (16)	0.84251 (11)	0.0461 (3)	
H2	0.3769	0.1931	0.9095	0.069*	

### Atomic displacement parameters (Å^2^)

**Table:**

	*U*^11^	*U*^22^	*U*^33^	*U*^12^	*U*^13^	*U*^23^
C1	0.0392 (10)	0.0326 (9)	0.0423 (10)	−0.0051 (7)	0.0017 (8)	0.0095 (8)
C2	0.0299 (8)	0.0272 (8)	0.0294 (8)	−0.0059 (7)	−0.0023 (6)	0.0018 (6)
C3	0.0241 (8)	0.0242 (8)	0.0282 (8)	−0.0041 (6)	−0.0011 (6)	−0.0001 (6)
C4	0.0250 (8)	0.0267 (8)	0.0281 (8)	−0.0051 (6)	−0.0004 (6)	0.0010 (6)
C5	0.0531 (13)	0.0762 (16)	0.0747 (16)	−0.0344 (12)	0.0045 (11)	0.0046 (12)
C6	0.0485 (11)	0.0488 (11)	0.0516 (12)	−0.0174 (9)	0.0003 (9)	−0.0063 (9)
C7	0.0298 (9)	0.0250 (8)	0.0454 (10)	−0.0028 (7)	0.0032 (7)	0.0040 (7)
C8	0.0248 (8)	0.0233 (8)	0.0269 (8)	−0.0032 (6)	0.0004 (6)	−0.0019 (6)
C9	0.0251 (8)	0.0230 (7)	0.0242 (8)	−0.0037 (6)	0.0001 (6)	−0.0011 (6)
C10	0.0237 (8)	0.0244 (8)	0.0270 (8)	−0.0028 (6)	−0.0012 (6)	−0.0002 (6)
N1	0.0247 (7)	0.0251 (7)	0.0313 (7)	−0.0019 (5)	0.0038 (5)	0.0036 (5)
N2	0.0293 (7)	0.0285 (7)	0.0311 (7)	−0.0062 (6)	0.0035 (5)	0.0031 (6)
N3	0.0257 (7)	0.0258 (7)	0.0321 (7)	−0.0030 (5)	0.0003 (5)	0.0039 (5)
N4	0.0258 (7)	0.0250 (7)	0.0419 (8)	0.0007 (5)	0.0089 (6)	0.0058 (6)
N6	0.0237 (7)	0.0234 (7)	0.0334 (7)	−0.0032 (5)	0.0008 (5)	0.0007 (5)
N7	0.0241 (7)	0.0228 (7)	0.0312 (7)	−0.0013 (5)	0.0016 (5)	0.0015 (5)
N8	0.0251 (7)	0.0239 (7)	0.0309 (7)	−0.0016 (5)	0.0009 (5)	0.0026 (5)
N9	0.0223 (7)	0.0269 (7)	0.0478 (9)	0.0005 (5)	0.0038 (6)	0.0078 (6)
N10	0.0237 (7)	0.0253 (7)	0.0411 (8)	−0.0017 (5)	0.0039 (6)	0.0054 (6)
O1	0.0477 (8)	0.0614 (9)	0.0398 (7)	−0.0274 (7)	0.0112 (6)	−0.0083 (6)
C11	0.0513 (12)	0.0746 (15)	0.0606 (14)	−0.0303 (11)	0.0095 (10)	−0.0034 (11)
C12	0.0453 (11)	0.0601 (13)	0.0463 (11)	−0.0190 (10)	0.0023 (9)	−0.0044 (9)
N5	0.0260 (7)	0.0298 (7)	0.0455 (8)	0.0005 (6)	0.0105 (6)	0.0072 (6)
O2	0.0461 (8)	0.0581 (8)	0.0391 (7)	−0.0241 (6)	0.0010 (6)	0.0112 (6)

### Geometric parameters (Å, °)

**Table:**

C1—C2	1.494 (2)	C8—N7	1.3327 (19)
C1—H1A	0.9600	C9—N10	1.3300 (19)
C1—H1B	0.9600	C9—N8	1.3471 (19)
C1—H1C	0.9600	C9—N7	1.3571 (19)
C2—N3	1.327 (2)	C10—N9	1.3364 (19)
C2—N2	1.340 (2)	C10—N8	1.3459 (19)
C3—N4	1.332 (2)	C10—N6	1.3580 (19)
C3—N1	1.3466 (19)	N4—H4A	0.8600
C3—N3	1.3576 (19)	N4—H4B	0.8600
C4—N5	1.332 (2)	N9—H9A	0.8600
C4—N1	1.346 (2)	N9—H9B	0.8600
C4—N2	1.355 (2)	N10—H10A	0.8600
C5—C6	1.490 (3)	N10—H10B	0.8600
C5—H5A	0.9600	O1—H1	0.8200
C5—H5B	0.9600	C11—C12	1.482 (3)
C5—H5C	0.9600	C11—H11A	0.9600
C6—O1	1.417 (2)	C11—H11B	0.9600
C6—H6A	0.9700	C11—H11C	0.9600
C6—H6B	0.9700	C12—O2	1.415 (2)
C7—C8	1.494 (2)	C12—H12A	0.9700
C7—H7A	0.9600	C12—H12B	0.9700
C7—H7B	0.9600	N5—H5D	0.8600
C7—H7C	0.9600	N5—H5E	0.8600
C8—N6	1.3285 (19)	O2—H2	0.8200
			
C2—C1—H1A	109.5	N10—C9—N7	116.38 (13)
C2—C1—H1B	109.5	N8—C9—N7	124.41 (13)
H1A—C1—H1B	109.5	N9—C10—N8	118.57 (13)
C2—C1—H1C	109.5	N9—C10—N6	116.26 (13)
H1A—C1—H1C	109.5	N8—C10—N6	125.17 (13)
H1B—C1—H1C	109.5	C4—N1—C3	114.56 (13)
N3—C2—N2	125.90 (14)	C2—N2—C4	114.89 (13)
N3—C2—C1	117.42 (14)	C2—N3—C3	114.50 (13)
N2—C2—C1	116.68 (14)	C3—N4—H4A	120.0
N4—C3—N1	117.82 (13)	C3—N4—H4B	120.0
N4—C3—N3	116.89 (13)	H4A—N4—H4B	120.0
N1—C3—N3	125.28 (13)	C8—N6—C10	114.35 (12)
N5—C4—N1	117.59 (14)	C8—N7—C9	114.99 (12)
N5—C4—N2	117.68 (13)	C10—N8—C9	114.81 (12)
N1—C4—N2	124.72 (14)	C10—N9—H9A	120.0
C6—C5—H5A	109.5	C10—N9—H9B	120.0
C6—C5—H5B	109.5	H9A—N9—H9B	120.0
H5A—C5—H5B	109.5	C9—N10—H10A	120.0
C6—C5—H5C	109.5	C9—N10—H10B	120.0
H5A—C5—H5C	109.5	H10A—N10—H10B	120.0
H5B—C5—H5C	109.5	C6—O1—H1	109.5
O1—C6—C5	109.36 (17)	C12—C11—H11A	109.5
O1—C6—H6A	109.8	C12—C11—H11B	109.5
C5—C6—H6A	109.8	H11A—C11—H11B	109.5
O1—C6—H6B	109.8	C12—C11—H11C	109.5
C5—C6—H6B	109.8	H11A—C11—H11C	109.5
H6A—C6—H6B	108.3	H11B—C11—H11C	109.5
C8—C7—H7A	109.5	O2—C12—C11	114.80 (17)
C8—C7—H7B	109.5	O2—C12—H12A	108.6
H7A—C7—H7B	109.5	C11—C12—H12A	108.6
C8—C7—H7C	109.5	O2—C12—H12B	108.6
H7A—C7—H7C	109.5	C11—C12—H12B	108.6
H7B—C7—H7C	109.5	H12A—C12—H12B	107.5
N6—C8—N7	126.22 (13)	C4—N5—H5D	120.0
N6—C8—C7	117.53 (13)	C4—N5—H5E	120.0
N7—C8—C7	116.25 (13)	H5D—N5—H5E	120.0
N10—C9—N8	119.21 (13)	C12—O2—H2	109.5
			
N5—C4—N1—C3	−177.19 (14)	N7—C8—N6—C10	−0.8 (2)
N2—C4—N1—C3	3.9 (2)	C7—C8—N6—C10	178.93 (13)
N4—C3—N1—C4	178.53 (14)	N9—C10—N6—C8	−178.84 (13)
N3—C3—N1—C4	−0.9 (2)	N8—C10—N6—C8	2.1 (2)
N3—C2—N2—C4	−0.2 (2)	N6—C8—N7—C9	−1.2 (2)
C1—C2—N2—C4	178.97 (14)	C7—C8—N7—C9	179.03 (13)
N5—C4—N2—C2	177.66 (14)	N10—C9—N7—C8	−177.94 (13)
N1—C4—N2—C2	−3.4 (2)	N8—C9—N7—C8	2.4 (2)
N2—C2—N3—C3	2.9 (2)	N9—C10—N8—C9	179.85 (13)
C1—C2—N3—C3	−176.34 (14)	N6—C10—N8—C9	−1.1 (2)
N4—C3—N3—C2	178.33 (14)	N10—C9—N8—C10	179.00 (13)
N1—C3—N3—C2	−2.3 (2)	N7—C9—N8—C10	−1.3 (2)

### Hydrogen-bond geometry (Å, °)

**Table:**

*D*—H···*A*	*D*—H	H···*A*	*D*···*A*	*D*—H···*A*
N4—H4B···N7^i^	0.86	2.11	2.9666 (18)	171
N5—H5D···N8^ii^	0.86	2.19	3.0132 (19)	159
N5—H5E···O2^iii^	0.86	2.29	3.0071 (19)	142
N10—H10A···O2^iv^	0.86	2.10	2.9337 (18)	163
O2—H2···O1^v^	0.82	1.90	2.7185 (18)	174

Symmetry codes: (i) *x*−1, *y*, *z*; (ii) *x*, *y*−1, *z*; (iii) −*x*+1, −*y*, −*z*+1; (iv) −*x*+1, −*y*+1, −*z*+1; (v) *x*, *y*, *z*+1.
